# Spatiotemporal and gender‐specific parasitism in two species of gobiid fish

**DOI:** 10.1002/ece3.4151

**Published:** 2018-05-20

**Authors:** Anssi Karvonen, Kai Lindström

**Affiliations:** ^1^ University of Jyvaskyla, Department of Biological and Environmental Science Jyvaskyla Finland; ^2^ Åbo Akademi University Environmental and Marine Biology Turku Finland

**Keywords:** host–parasite interaction, mate choice, parasite community, sexual selection, virulence

## Abstract

Parasitism is considered a major selective force in natural host populations. Infections can decrease host condition and vigour, and potentially influence, for example, host population dynamics and behavior such as mate choice. We studied parasite infections of two common marine fish species, the sand goby (*Pomatoschistus minutus*) and the common goby (*Pomatoschistus microps*), in the brackish water Northern Baltic Sea. We were particularly interested in the occurrence of parasite taxa located in central sensory organs, such as eyes, potentially affecting fish behavior and mate choice. We found that both fish species harbored parasite communities dominated by taxa transmitted to fish through aquatic invertebrates. Infections also showed significant spatiotemporal variation. Trematodes in the eyes were very few in some locations, but infection levels were higher among females than males, suggesting differences in exposure or resistance between the sexes. To test between these hypotheses, we experimentally exposed male and female sand gobies to infection with the eye fluke *Diplostomum pseudospathaceum*. These trials showed that the fish became readily infected and females had higher parasite numbers, supporting higher susceptibility of females. Eye fluke infections also caused high cataract intensities among the fish in the wild. Our results demonstrate the potential of these parasites to influence host condition and visual abilities, which may have significant implications for survival and mate choice in goby populations.

## 1 INTRODUCTION

Parasitism is a potent source of selection in natural host populations and has recently been suggested to play a role in processes such as maintenance of sexual reproduction (Jokela, Dybdahl, & Lively, 2009; King, Delph, Jokela, & Lively, 2009) and divergence of host populations (Eizaguirre, Lenz, Kalbe, & Milinski, 2012; Karvonen & Seehausen, 2012). Indeed, several parasite taxa impair host condition through depletion of resources, tissue damage, and manipulation of host behavior (Barber, Hoare, & Krause, 2000; Barber & Svensson, 2003; Hafer & Milinski, 2016; Jokela, Taskinen, Mutikainen, & Kopp, 2005; Karvonen, Seppälä, & Valtonen 2004a; Moore, 2002; Seppälä, Liljeroos, Karvonen, & Jokela, 2008) and thus have severe implications for host fitness. The risk of parasitism is often structured both spatially and temporally because of spatial aggregation of infected individuals and parasite intermediate hosts (Byers, Blakeslee, Linder, Cooper, & Maguire, 2008; Faltýnková, Valtonen, & Karvonen, 2008; Jokela & Lively, 1995; Karvonen, Cheng, & Valtonen, 2005), and seasonal changes in release of parasite infective stages (Karvonen, Seppälä, & Valtonen 2004b; Taskinen, Valtonen, & Mäkelä, 1994). Consequently, the impact of parasites can also vary among host populations and, if persistent, such differences can potentially create different selection pressures for host individuals living in these populations (Karvonen & Seehausen, 2012).

Several host species of parasites express secondary sexual characteristics through which they can advertise their vigour, as well as resistance to parasites (Hamilton & Zuk, 1982). Often these signals are visually perceived ornaments such as long tails or bright coloration, and commonly displayed by males. For example, the connection between the expression of sexual ornaments and parasitism has been demonstrated in many species of birds (Hõrak, Ots, Vellau, Spottiswoode, & Pape Møller, 2001; McGraw & Hill, 2000; Thompson, Hillgarth, Leu, & McClure, 1997) and fish (Barber, Arnott, Braithwaite, Andrew, & Huntingford, 2001; Houde & Torio, 1992; Maan, van der Spoel, Jimenez, van Alphen, & Seehausen, 2006). Overall, current evidence strongly suggests that male sexual ornaments could signal resistance to parasitism. However, reduction in host vision could impair the ability of individuals to perceive sexual signals. For example, it has been shown in cichlid fishes that visually perceived sexual signals advertised through male coloration can be blurred because of increased water turbidity, resulting in hybridization of species following the relaxation of color‐based sexual selection (Seehausen, van Alphen, & Witte, 1997). Similarly, parasites found in the key sensory organs, such as the eyes, could impair host vision and the ability to perceive visual cues from potential mates (Karvonen & Seehausen, 2012). In species where sexual selection is based on males displaying secondary sexual characteristics, the ability of females to judge male quality when infected with such parasites could be compromised. However, while mating decisions are known to be influenced by general condition (Cotton, Small, & Pomiankowski, 2006) and also parasite infections (Lopez, 1999; Mazzi, 2004; Pfennig & Tinsley, 2002; Poulin & Vickery, 1996) of the choosier sex, the potential of parasites directly interfering with sexual selection operating through visual signals perceived by females has remained virtually unexplored. Here, we explore parasitism and particularly the infections in the eyes of gobiid fishes where females actively choose males based on secondary sexual characteristics.

Gobies (Gobiidae) are abundant fish species living in marine and brackish water habitats around the world. Five species of gobies inhabit the Baltic Sea, two of which, the sand goby (*Pomatoschistus minutus*, Pallas 1770) and the common goby (*Pomatoschistus microps*, Krøyer 1838), are the most common. During the reproductive period in early summer, males build nests where they attract females to spawn using secondary sexual traits. In the sand goby, for instance, these include a bright blue spot on the first dorsal fin and the size of the fin itself, as well as specific courtship behavior (Forsgren, 1992; Lindström, St. Mary, & Pampoulie, 2006). Also, the size of the nest is one of the key determinants of reproductive success of a male because the most fecund females cannot lay all their eggs in small nests (Lindström, 1992). All these characteristics of male quality are perceived visually by females.

Previous studies have shown that gobies also harbor a diverse parasite fauna. For example, Baltic gobies can host four to 22 species of parasites depending on the host species and sampling time (Zander, 2003). Further, Zander (2005) reported that the parasite communities were often most diverse in autumn with very few species present in the spring. Studies have also reported higher parasite infection among wild‐caught female sand gobies compared to males (Van Damme & Ollevier, 1994), suggesting higher exposure and/or susceptibility of females. Finally, behavioral trials have shown that infection of male sand gobies with macroparasites in the body cavity and on the fins and skin did not affect male dominance or female mate choice (Barber, 2002). However, Barber (2002) also found that infection intensity of *Gyrodactylus* monogeneans had a negative effect on the development of the dorsal fin size, a potential secondary sexual trait of the sand goby.

We explored parasite infections of the sand goby and the common goby in the Baltic Sea by conducting a replicated sampling campaign of male and female fish to capture spatiotemporal variation and possible gender differences in parasitism. We were particularly interested in variation in infection of parasites inhabiting sensory organs of fish that could show the potential of parasite‐induced changes in conditions of sexual selection and mate choice among the sampling locations. Similarly, infections could influence overwinter survival of the fish and result in lower infection levels in the early summer. Furthermore, to explain patterns of infection of the parasites in the wild, we exposed male and female sand gobies to controlled experimental infection from trematode eye flukes in the laboratory. Differences in the abundance of these parasites between male and female fish under similar level of exposure would be consistent with the idea of differences in susceptibility between the sexes.

## 2 MATERIALS AND METHODS

### 2.1 Sampling of gobies

Sand gobies and common gobies were sampled from three locations in the proximity of the Tvärminne Zoological station, southern Finland. The first location was next to the station (referred here to as “Station”; 59°50′41″N, 23°14′58″E), the second ca. 500 m from the Station (Långholmen; 59°50′48″N, 23°15′12″E), and the third farther out to the sea ca. 4 km from the Station (Vargskär; 59°49′24″N, 23°08′38″E). All locations had a bottom substrate of sand or soft mud with little or no vegetation. Sampling was conducted three times, June 2014, June 2015, and October 2015, to capture both spatial and temporal variation in infections. At each location, gobies were caught from a depth of 0.5–1 m using a seine net. However, sampling campaigns at some of the locations were unsuccessful at times (Table 1) due to complete absence of fish. Fish were brought alive to the laboratory, euthanized, sexed, measured for length (mm), and inspected fresh for infections on fins (right pectoral fin), gills, eyes, and internal organs under a microscope. Parasites were identified at genus or species level when possible. Prevalence (proportion of fish infected, %) and mean abundance (mean parasite number per fish) were calculated for each parasite taxa. In addition, fish eye lenses were studied before dissection for coverage of cataracts caused by *Diplostomum* spp. eye flukes using slit‐lamp (Kowa SL‐15) microscopy (Karvonen et al., 2004a). Cataracts were scored as 10%, 20%,…,100% coverage of the lens area, which correlates with the deleterious effects of the parasites on fish (Karvonen & Seppälä, 2008; Seppälä, Karvonen, & Valtonen, 2005) and thus provides an indirect measure of parasite‐induced effects on the host. Differences in total parasite abundance between the locations and sampling times were analyzed using GLMs with negative binomial distribution and log link function. Abundances of the parasite taxa *Trichodina* sp. and *Gyrodactylus* sp. (see Results) were excluded from the analyses as they were studied only from one pectoral fin.

**Table 1:** Prevalence (% fish infected) and mean abundance (number of parasites per fish ± *SE*) of the parasite taxa detected in sand gobies captured from three locations in the Northern Baltic Sea in June 2014–October 2015

Parasite	June 2014			June 2015			October 2015		
	Station *n* = 29	Långholmen *n* = 30	Vargskär *n* = 31	Station *n* = 30	Långholmen *n* = 30	Vargskär *n* = 30	Station *n* = 0	Långholmen *n* = 6	Vargskär *n* = 14
*Trichodina* sp.	90 11.2 ± 3.4	86.7 2.9 ± 0.4	67.7 2.6 ± 0.9	96.7 24.6 ± 6.6	96.7 14.0 ± 2.8	66.7 5.6 ± 1.8		16.7 0.2 ± 0.2	57.1 1.7 ± 0.7
*Gyrodactylus* sp.	0.0	10 0.1 ± 0.1	0.0	10 0.1 ± 0.1	3.3 0.03 ± 0.03	10 0.1 ± 0.1		0.0	57.1 0.9 ± 0.4
*Diplostomum* spp.	0.0	6.7 0.07 ± 0.05	3.2 0.03 ± 0.03	26.7 0.4 ± 0.1	23.3 0.2 ± 0.1	30 0.4 ± 0.1		16.7 0.2 ± 0.2	78.6 2.8 ± 11.3
*Diplostomum baeri*	3.4 0.07 ± 0.07	0.0	0.0	6.7 0.07 ± 0.05	3.3 0.03 ± 0.03	0.0		0.0	21.4 0.4 ± 0.2
*Apatemon* sp.	31 0.4 ± 0.1	33.3 0.8 ± 0.3	9.7 0.1 ± 0.1	40 1.0 ± 0.3	30 0.5 ± 0.2	40 0.9 ± 0.3		16.7 0.2 ± 0.2	64.3 2.9 ± 1.6
*Cryptocotyle* sp.	56 0.9 ± 0.2	80 2.9 ± 0.5	80.6 5.6 ± 1.0	50 1.2 ± 0.3	26.7 0.5 ± 0.2	70 6.2 ± 1.6		0.0	71.4 2.2 ± 0.5
*Sphaerostomum* sp.	0.0	6.7 0.07 ± 0.05	0.0	0.0	0.0	0.0		0.0	0.0
*Proteocephalus* sp.	13.8 0.2 ± 0.1	13.3 0.1 ± 0.1	6.5 0.1 ± 0.1	3.3 0.03 ± 0.03	3.3 0.03 ± 0.03	3.3 0.03 ± 0.03		0.0	0.0
*Camallanus lacustris*	3.4 0.03 ± 0.03	3.3 0.07 ± 0.07	0.0	0.0	0.0	0.0		0.0	0.0
Unidentified nematode	10.3 0.1 ± 0.1	16.7 0.2 ± 0.1	16.1 0.2 ± 0.1	6.7 0.07 ± 0.05	0.0	6.7 0.07 ± 0.05		0.0	7.1 0.07 ± 0.07
*Echinorhynchus gadii*	6.9 0.1 ± 0.1	0.0	0.0	0.0	0.0	0.0		0.0	0.0
*Neoechinorhynchus rutili*	6.9 0.07 ± 0.05	0.0	6.5 0.2 ± 0.1	0.0	6.7 0.1 ± 0.1	6.7 0.1 ± 0.1		0.0	0.0

### 2.2 Experimental exposure

**Figure 1:**
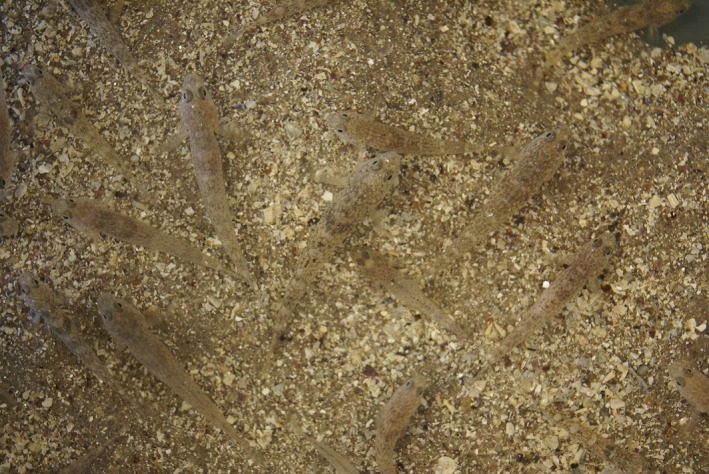
Female sand gobies (*Pomatoschistus minutus*). Photograph by Kai Lindström

Experimental exposure of sand gobies (Figure 1) was conducted in three containers each with 6 l of water (16°C) taken from the Baltic Sea, continuous aeration, and 10 fish (five females and five males) captured 1 week earlier from the Vargskär sampling location, totaling 30 fish (mean length: 48.0 ± 2.0 mm [females], 53.5 ± 1.9 mm [males]). Before the experiment, the fish had been housed in replicated large stock aquaria supplied with a continuous flow of fresh seawater and fed with live mysiid shrimp and frozen Chironomidae larvae ad libitum. In addition to the three exposure containers, one container with 10 fish and an even sex ratio served as the unexposed control. This was used to record possible infections resulting from parasite infective stages present in the water, if any, and those that had taken place recently in the wild before the fish were caught, which could not be separated from those resulting from the experimental exposure (see below). Each of the three infection containers then received a total dose of 750 *D. pseudospathaceum* cercariae (75 cercariae per fish) that had been released by five infected *Lymnaea stagnalis* snails collected from Lake Vuojärvi, Central Finland. Note that there is no detectable population genetic structure in these parasites across Finland (Louhi, Karvonen, Rellstab, & Jokela, 2010), which is why parasite origin was unlikely to affect the results. The snails were allowed to produce cercariae for 2 hr in 2 dl of water (20°C). Suspensions of the snails were then combined, and the cercarial density was estimated by taking ten 1 ml samples. Water in the containers was regularly mixed during the first hour of exposure to ensure equal exposure of all individuals to the parasite. The fish were maintained in these conditions for 18 hr, which is sufficient time for the parasites to reach the eye lenses in a small fish (Louhi, Sundberg, Jokela, & Karvonen, 2015). There was no mortality of fish during or after the exposure. All fish were then euthanized and studied for the number of parasites in the eye lenses. Older infections originating from the wild and those originating from the experimental exposure could be distinguished based on the size of the parasite metacercariae. All experimental procedures were in accordance with the ethical standards of the Finnish Regional State Administrative Agency and conducted under license (License code: ESAVI/4706/04.10.07/2015). Data were analyzed using ANCOVA with fish sex as a fixed factor and container as a random factor to account for dependency among fish exposed in the same container. Fish length was used as a covariate. All analyses were conducted using SPSS 24 statistical package.

## 3 RESULTS

**Figure 2:**
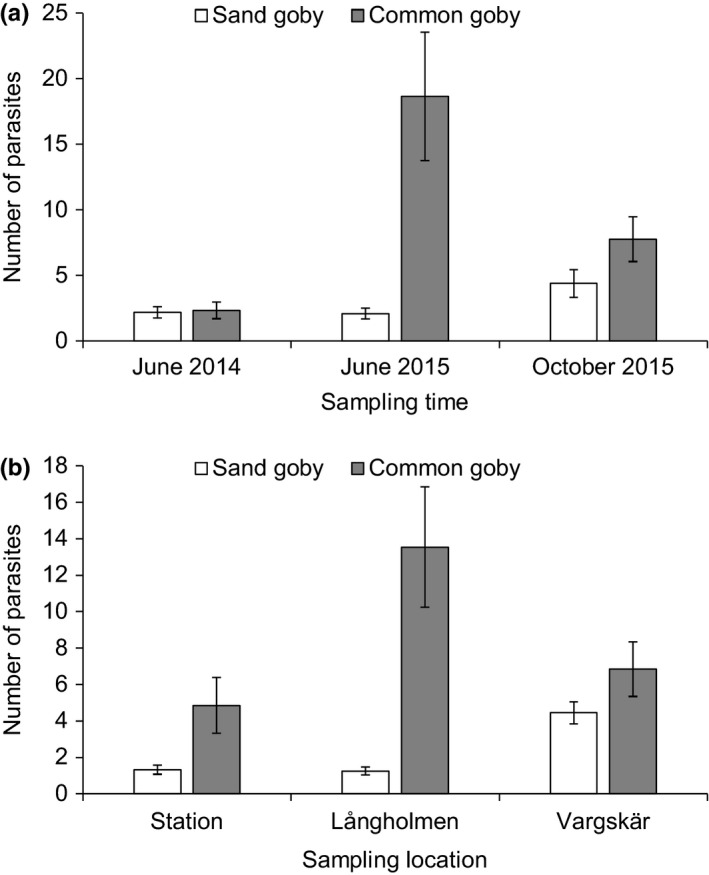
Estimated mean total parasite abundance (±*SE*) in sand gobies and common gobies captured at three sampling times (a, sampling locations combined) and from three sampling locations (b, sampling times combined) in the Northern Baltic Sea. Estimates are from GLM models. Numbers of fish studied are indicated in Table 1

In total, 12 parasite taxa were detected among the 200 sand gobies and 168 common gobies examined, including five trematodes, two nematodes, two acanthocephalans, one cestode, one monogenean, and one protozoan (Table 1, Table 2). All parasite taxa were observed in both fish species, except for the acanthocephalan *Echinorhynchus gadii*, which was observed only in the sand goby. The mean number of parasite taxa per fish was significantly higher in the sand goby (2.23 ± 0.1, all numbers indicate mean ± *SE*, range 0–7) than in the common goby (1.78 ± 0.1, range 0–4) (*t* test: *t*_354_ = 3.76, *p* < .001; locations and sampling times combined). In both fish species, the protozoan *Trichodina* sp. was the most prevalent and abundant parasite taxa with prevalence ranging between 16.7% and 96.7% in the sand goby and 0%–100% in the common goby, depending on the location and sampling time (Table 1, Table 2). Among the macroparasites, trematodes *Cryptocotyle* sp., *Apatemon* sp., and *Diplostomum* spp. were most commonly observed. Mean total parasite abundance was significantly higher in the common goby compared to the sand goby (GLM: Wald = 11.76, *p* < .001; sampling locations combined) while this depended on the sampling time (Wald = 30.65, *p* < .001 (sampling time); Wald = 32.86, *p* < .001 (species × sampling time); Figure 2). There were also significant differences in parasite abundance among the locations so that Långholmen and Vargskär locations had the highest parasite abundances (GLM: Wald = 14.25, *p* = .001; sampling times combined) while this again depended on the fish species (Wald = 28.01, *p* < .001 (species); Wald = 32.63, *p* < .001 (species × sampling location); Figure 2).

**Figure 3:**
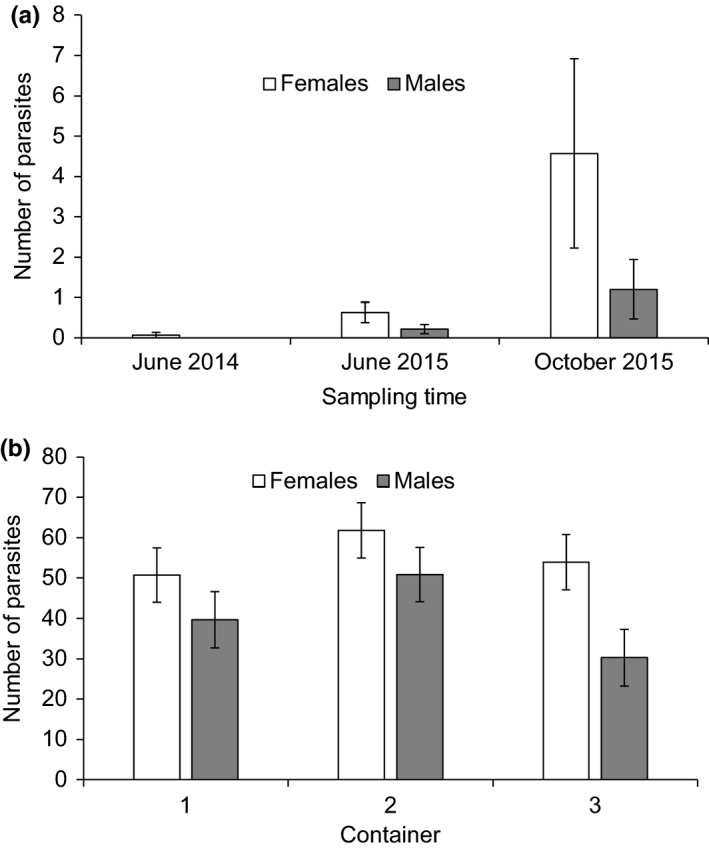
Mean abundance of *Diplostomum* spp. eye flukes (±*SE*) in female and male sand gobies captured from Vargskär at three sampling times (a). Estimated mean abundance of *D. pseudospathaceum* eye flukes (±*SE*) from the ANCOVA model in female and male sand gobies exposed to experimental parasite infection in three containers in the laboratory (b)

**Figure 4:**
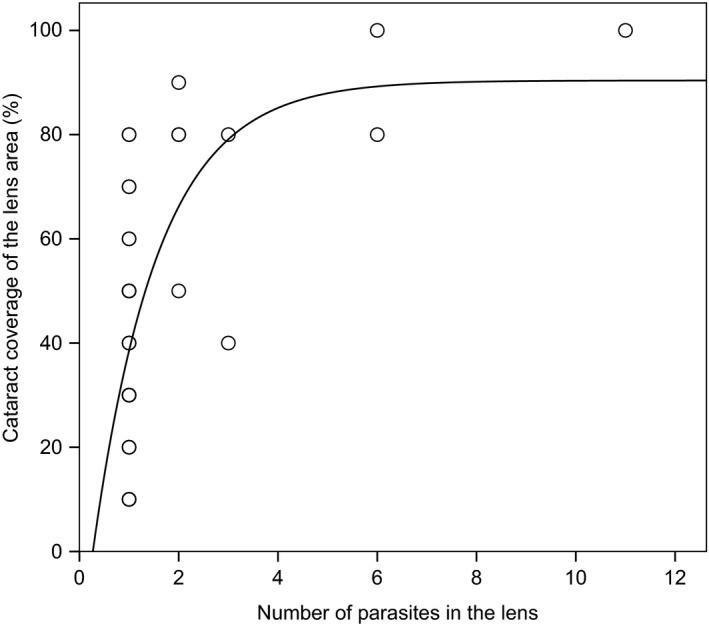
Relationship between the abundance of *Diplostomum* spp. eye flukes and the coverage of parasite‐induced cataracts in eye lenses of sand gobies (data pooled across sampling locations and sampling times). Black line shows the fit of the nonlinear regression model [cataract coverage = 90.4 + −111.1*exp(−0.76*parasites), *R* ^2^ = .41, *F*_3,35 _= 72.5, *p* < .0001]

Total parasite abundance was not different between males and females in either fish species (Wald = 1.05, *p* = .305 (sex); Wald = 1.98, *p* = .159 (species × sex); immature fish excluded, sampling locations and sampling times combined). However, a more detailed analysis of the infections of *Diplostomum* eye fluke in Vargskär showed that females harbored significantly higher abundances of these parasites compared to males (GLM: Wald = 4.94, *p* = .026 (sex); Wald = 30.64, *p* < .001 (sampling time); Figure 3a). Infections of eye flukes also caused significant cataract coverage in the eye lenses of sand gobies. Cataract coverage increased with parasite abundance so that the coverage rapidly approached 100% when there was more than one parasite per lens. The relationship was captured by a steep asymptotic curve (Figure 4). However, there was no difference in cataracts caused by a given parasite abundance between the male and female sand gobies (*t* test on residual cataract coverage from the nonlinear regression: *t*_36_ = 0.228, *p* = .821). While this suggests similar susceptibility to parasite‐inflicted damage between the sexes, eyes of female sand gobies in Vargskär nevertheless showed higher average cataracts than males because of their higher parasite abundances (*t* test: *t*_82_ = 2.082, *p* = .040). No cataracts were observed in uninfected eye lenses.

Experimental exposure of sand gobies from Vargskär to *D. pseudospathaceum* infection indicated that all fish became readily infected with the parasite. The mean parasite abundance per fish increased with body size (ANCOVA: *F*_1,23_ = 15.60, *p* < .001) and ranged from 43.0 ± 6.0 to 52.6 ± 5.8 depending on the container (*F*_2,2.1_ = 4.06, *p* = .193 (container); Figure 3b). Similarly to the field data (see above), there was a significant difference in parasite abundance between the sexes so that females were more heavily infected (*F*_1,3.0_ = 10.56, *p* = .047 (sex); *F*_2,23_ = 0.58, *p* = .568 (sex × container); Figure 3b). This suggests higher susceptibility of females to infection. New or recent infections were not detected among the 10 unexposed control fish indicating that there was no natural exposure from the water during the experiment and that the fish had not been recently infected in the wild. Four of the 30 exposed fish and one of the 10 control fish harbored fully developed *Diplostomum* metacercariae, likely as a result of infection in the wild in the previous summer.

## 4 DISCUSSION

Spatial and temporal variation in parasitism can have significant implications for host populations by underlying variability in influence of parasites on the condition and reproduction of individuals (Lefevre et al., 2009; Tompkins, Dunn, Smith, & Telfer, 2011; Wood et al., 2007). This is particularly clear with infections that can cause detrimental effects on the hosts, such as those located in the central sensory organs. Moreover, hosts species in which such effects can come about already at low parasite numbers may suffer the most notable consequences. We examined parasite infections of two species of gobiid fishes in the Northern Baltic Sea. We scored the entire macroparasite community in these fishes and specifically targeted eye flukes of the genus *Diplostomum* that are known to cause loss of vision in several fish species, with the effects likely being most prominent in smaller fish species with the smallest eye lenses. We found significant spatiotemporal variation in infections as well as differences between the fish species so that the common gobies were more heavily infected in most cases. While there was no effect of host sex on the infections overall, female sand gobies were more heavily infected with eye flukes at the sampling location with the highest infection. An experimental exposure of the fish indicated that the difference between the sexes was at least partly explained by the higher susceptibility of females to infection.

**Table 2:** Prevalence (% fish infected) and mean abundance (number of parasites per fish ±* SE*) of the parasite taxa detected in common gobies captured from three locations in the Northern Baltic Sea in June 2014–October 2015

Parasite	June 2014			June 2015			October 2015		
	Station *n* = 30	Långholmen *n* = 24	Vargskär *n* = 0	Station *n* = 30	Långholmen *n* = 30	Vargskär *n* = 0	Station *n* = 0	Långholmen *n* = 24	Vargskär *n* = 30
*Trichodina* sp.	100 18.0 ± 5.7	95.8 32 ± 0.5		96.7 16.8 ± 4.2	100 15.3 ± 2.9			0.0	60.0 0.9 ± 0.2
*Gyrodactylus* sp.	3.3 0.03 ± 0.03	50.0 0.7 ± 0.2		16.7 0.5 ± 0.3	3.3 0.03 ± 0.03			0.0	6.7 0.07 ± 0.05
*Diplostomum* spp.	0.0	0.0		3.3 0.03 ± 0.03	30.0 0.4 ± 0.1			83 0.1 ± 0.1	13.3 0.1 ± 0.1
*Diplostomum baeri*	0.0	0.0		0.0	3.3 0.03 ± 0.03			0.0	0.0
*Apatemon* sp.	3.3 0.07 ± 0.07	83 0.1 ± 0.1		30 0.3 ± 0.1	13.3 0.1 ± 0.1			83 0.1 ± 0.1	13.3 0.2 ± 0.1
*Cryptocotyle* sp.	16.7 0.5 ± 0.4	58.3 2.0 ± 0.6		63.3 4.2 ± 1.4	76.6 13.1 ± 3.5			45.8 3.7 ± 1.3	73.3 3.9 ± 0.8
*Sphaerostomum* sp.	3.3 0.03 ± 0.03	0.0		0.0	0.0			0.0	0.0
*Proteocephalus* sp.	3.3 0.03 ± 0.03	0.0		0.0	13.3 0.1 ± 0.1			0.0	3.3 0.03 ± 0.03
*Camallanus lacustris*	6.7 0.07 ± 0.05	0.0		0.0	0.0			0.0	0.0
Unidentified nematode	6.7 0.07 ± 0.05	4.2 0.04 ± 0.04		0.0	3.3 0.03 ± 0.03			42 0.04 ± 0.04	0.0
*Echinorhynchus gadii*	0.0	0.0		0.0	0.0			0.0	0.0
*Neoechinorhynchus rutili*	0.0	4.2 0.04 ± 0.04		0.0	0.0			0.0	0.0

Spatiotemporal variation in parasitism is a common feature of most host–parasite interactions, including parasitic infections of fish in freshwater (Marcogliese, Gendron, Plante, Fournier, & Cyr, 2006; de Roij & MacColl, 2012) and in sea (Grutter, 1998; Sikkel, Nemeth, McCammon, & Williams, 2009). We found that parasite infections of the two goby species followed similar patterns. First, infection abundances were clearly different between the locations, particularly in the common goby, and mainly driven by the trematode *Cryptocotyle* sp. This could reflect, for example, spatial variation in infection prevalence in the first intermediate snail hosts of the parasite, which is commonly observed also in other trematode systems (Faltýnková et al., 2008; Jokela & Lively, 1995). Second, with few exceptions, directly transmitted parasites (*Trichodina* sp. and *Gyrodactylus* sp.) were clearly more prevalent in the early summer compared to autumn, reflecting the temperature‐driven replication of these parasites (Bagge & Valtonen, 1999; Halmetoja, Valtonen, & Taskinen, 1992; Koskivaara, Valtonen, & Prost, 1991; Rintamäki‐Kinnunen & Valtonen, 1997). Third, trophically transmitted parasites (cestodes, nematodes, and acanthocephalans) showed sporadic occurrence at low numbers, suggesting that the gobies unlikely are the primary fish hosts for these parasites in this system. Fourth, many of the larval trematodes, particularly in sand gobies from the Vargskär location with the highest infection, tended to be more abundant in autumn compared to early summer (Table 1). This well reflects the typical accumulation of trematodes in their intermediate hosts during summer months (Faltýnková, Karvonen, & Valtonen, 2011; Karvonen, Hudson, Seppälä, & Valtonen, 2004; Karvonen et al., 2004a,b). The lower abundance in the early summer is also consistent with the idea that the most heavily infected individuals may be lost from the population during winter, while our data were too few to test this properly. For example, *Diplostomum* infections and cataracts comparable to this study in the eye lenses are known to cause serious fitness consequences in fish (Crowden & Broom, 1980; Karvonen & Seppälä, 2008; Seppälä et al., 2005), supporting a possibility of parasite‐driven population effects (Marcogliese, Compagna, Bergeron, & McLaughlin, 2001). Overall, broad lines of the spatial and temporal variation of parasitism in this system are in accordance with earlier findings of parasite infections in gobies in the Baltic Sea (Zander, 2003, 2005; Zander & Kesting, 1998; Zander, Strohbach, & Groenewold, 1993).

We also found a distinct difference between male and female sand gobies in the abundance of eye fluke infection so that females were more heavily infected both in the field and in the experimental exposure. This is in contrast with the general pattern of higher infestation in males across a range of host–parasite systems (Klein, 2004; Poulin, 1996), although few studies have reported higher parasite infections also in females, for example, in guppies (Richards, van Oosterhout, & Cable, 2010; Stephenson, van Oosterhout, Mohammed, & Cable, 2015; Tadiri, Scott, & Fussmann, 2016), gobies (Van Damme & Ollevier, 1994) and coral reef fish (Sikkel, Fuller, & Hunte, 2000). One reason for the sex difference in this system could be that males and females in the field are differently exposed to the parasite cercariae. Our sampling time in early summer coincided with the breeding season of gobies (Hesthagen, 1977; Lindström, 1998; Nyman, 1953) when males are defending nests and eggs, and remain mostly stationary. On the other hand, females are actively swimming around in search of spawning and feeding opportunities, which could increase their exposure to the parasite cercariae (see also Sikkel et al. (2000)). However, our exposure experiment showed that the sex difference in infections is not merely due to behavioral differences, but that females are also more susceptible to infection than males. This is interesting as it contradicts with the general trend of males being more susceptible to parasite infections because of sex hormones that can suppress immune function (Klein, 2004). The reason why such a sex‐specific infection pattern was observed only in Vargskär is currently unclear, but may be related to differences between the habitat types. For example, the sampling sites Station and Långholmen are more sheltered compared to the more exposed Vargskär, although this would suggest lower rather than higher infection in the latter. On the other hand, the population size of seagulls, terns, and merganserids, the definitive hosts for many trematodes including *Diplostomum*, may be larger at Vargskär than the other sites (Lindström & Ranta, 1992). This could enhance parasite life cycles locally (Marcogliese et al., 2001). However, details of the differences in infection processes need further work.

Our data also show that just one to two worms infecting an eye lens of small fish, such as gobies, can severely harm the lens (i.e., cataracts covering the majority of lens were formed at very low infection). This is because the size of the parasite metacercariae (and the damage they inflict per lens volume) likely remain relatively constant regardless of the lens size, but the size of the eye lens increases with fish size. Thus, in larger fish species, cataract coverage typically increases linearly with the parasite abundance and tens of parasites per lens may be required for high cataract intensities (Karvonen & Seppälä, 2008; Karvonen et al., 2004a), whereas even a low‐level infection is likely to severely impair the vision of a small fish (see also Owen, Barber, and Hart (1993)). During mating, female sand gobies visit several males (Forsgren, 1997; Lindström & Lehtonen, 2013) and base their mate choice on a range of visual cues (Forsgren, 1992; Lindström et al., 2006). As a consequence, attractive males reach high mating success compared to less attractive ones (Lindström & Seppä, 1996) and this nonrandom distribution of mating success results in sexual selection (Andersson, 1994; Emlen & Oring, 1977). This process, however, can be potentially affected by impaired visual ability of females. For example, decreased water clarity interferes with visual abilities of females and this has been invoked as an explanation for weakened sexual selection in turbid waters (Järvenpää & Lindström, 2004; Seehausen et al., 1997). Similarly, if the visual ability of females was hampered by *Diplostomum*, this could affect the way females can judge males and express their mating preferences, as they would not be able to detect male mating signals. Consequently, mating systems in areas of high infection risk for females could become more random and result in weakened sexual selection. Such a process could easily create spatial variation in the intensity of sexual selection and may contribute to preserving genetic variation in male secondary sexual traits. However, female preferences are also based on the quality of male parental care (Lindström et al., 2006; Pampoulie, Lindström, & St. Mary, 2004). Thus, it is possible that females in areas of high infection, on average, select males exhibiting lower quality care, which then results in lower offspring production. While our data are suggestive of the potential for such parasite‐induced changes in mate choice, these questions need to be tackled experimentally.

To conclude, spatiotemporal variation in parasitism among populations, as well as that between sexes, can determine to what extent host populations suffer fitness consequences of infections in terms of decreased survival and reproductive success. Several earlier studies have suggested that sexual characteristics used in mate choice can advertise resistance of an individual toward parasite infections. However, the alternative that parasite infections could influence this process by impairing the visual ability of one sex to perceive such characteristics is virtually unexplored. Our data show the potential for such changes in mate choice in small‐sized fish such as gobies, where visual abilities of females perceiving signals from males could deteriorate at very low infection intensities. However, we also suggest that such effects are likely to be different among host populations experiencing different levels of parasitism. Overall, relationships between spatiotemporal variation in infections, gender‐biased parasitism and mate choice form an interesting field for further experimental research.

## CONFLICT OF INTEREST

The authors declare that they have no conflict of interest.

## AUTHOR CONTRIBUTIONS

AK and KL conceived, designed, and performed the experiments. AK analyzed the data. AK and KL wrote the manuscript.

## References

[ece34151-bib-0001] Andersson, M. (1994). Sexual selection. Princeton, NJ: Princeton University Press.

[ece34151-bib-0002] Bagge, A. M. , & Valtonen, E. T. (1999). Development of monogenean communities on the gills of roach fry (*Rutilus rutilus*). Parasitology, 118, 479–487. https://doi.org/10.1017/S0031182099004011 10363281 10.1017/s0031182099004011

[ece34151-bib-0003] Barber, I. (2002). Parasites, mate‐male competition and female mate choice in the sand goby. Journal of Fish Biology, 61, 185–198.

[ece34151-bib-0004] Barber, I. , Arnott, S. A. , Braithwaite, V. A. , Andrew, J. , & Huntingford, F. A. (2001). Indirect fitness consequences of mate choice in sticklebacks: Offspring of brighter males grow slowly but resist parasitic infections. Proceedings of the Royal Society B‐Biological Sciences, 268, 71–76. https://doi.org/10.1098/rspb.2000.1331 10.1098/rspb.2000.1331 PMC1087602 12123300

[ece34151-bib-0005] Barber, I. , Hoare, D. , & Krause, J. (2000). Effects of parasites on fish behaviour: A review and evolutionary perspective. Reviews in Fish Biology and Fisheries, 10, 131–165. https://doi.org/10.1023/A:1016658224470

[ece34151-bib-0006] Barber, I. , & Svensson, P. A. (2003). Effects of experimental *Schistocephalus solidus* infections on growth, morphology and sexual development of female three‐spined sticklebacks, *Gasterosteus aculeatus* . Parasitology, 126, 359–367. https://doi.org/10.1017/S0031182002002925 12741515 10.1017/s0031182002002925

[ece34151-bib-0007] Byers, J. E. , Blakeslee, A. M. H. , Linder, E. , Cooper, A. B. , & Maguire, T. J. (2008). Controls of spatial variation in the prevalence of trematode parasites infecting a marine snail. Ecology, 89, 439–451. https://doi.org/10.1890/06-1036.1 18409433 10.1890/06-1036.1

[ece34151-bib-0008] Cotton, S. , Small, J. , & Pomiankowski, A. (2006). Sexual selection and condition‐dependent mate preferences. Current Biology, 16, R755–R765. https://doi.org/10.1016/j.cub.2006.08.022 16950102 10.1016/j.cub.2006.08.022

[ece34151-bib-0009] Crowden, A. E. , & Broom, D. M. (1980). Effects of the eyefluke, *Diplostomum spathaceum*, on the behavior of dace (*Leuciscus leuciscus*). Animal Behaviour, 28, 287–294. https://doi.org/10.1016/S0003-3472(80)80031-5

[ece34151-bib-0010] Eizaguirre, C. , Lenz, T. L. , Kalbe, M. , & Milinski, M. (2012). Divergent selection on locally adapted major histocompatibility complex immune genes experimentally proven in the field. Ecology Letters, 15, 723–731. https://doi.org/10.1111/j.1461-0248.2012.01791.x 22583762 10.1111/j.1461-0248.2012.01791.x PMC3440595

[ece34151-bib-0011] Emlen, S. T. , & Oring, L. W. (1977). Ecology, sexual selection, and the evolution of mating systems. Science, 197, 215–223. https://doi.org/10.1126/science.327542 327542 10.1126/science.327542

[ece34151-bib-0012] Faltýnková, A. , Karvonen, A. , & Valtonen, E. T. (2011). Establishment and interspecific associations in two species of *Ichthyocotylurus* (Trematoda) parasites in perch (*Perca fluviatilis*). Parasites & Vectors, 4, 85 https://doi.org/10.1186/1756-3305-4-85 21599910 10.1186/1756-3305-4-85 PMC3121694

[ece34151-bib-0013] Faltýnková, A. , Valtonen, E. T. , & Karvonen, A. (2008). Spatial and temporal structure of the trematode component community in *Valvata macrostoma* (Gastropoda, Prosobranchia). Parasitology, 135, 1691–1699. https://doi.org/10.1017/S0031182008005027 18992180 10.1017/S0031182008005027

[ece34151-bib-0014] Forsgren, E. (1992). Predation risk affects mate choice in a Gobiid fish. The American Naturalist, 140, 1041–1049. https://doi.org/10.1086/285455

[ece34151-bib-0015] Forsgren, E. (1997). Mate sampling in a population of sand gobies. Animal Behaviour, 53, 267–276. https://doi.org/10.1006/anbe.1996.0374

[ece34151-bib-0016] Grutter, A. S. (1998). Habitat‐related differences in the abundance of parasites from a coral reef fish: An indication of the movement patterns of *Hemigymnus melapterus* . Journal of Fish Biology, 53, 49–57.

[ece34151-bib-0017] Hafer, N. , & Milinski, M. (2016). Inter‐ and intraspecific conflicts between parasites over host manipulation. Proceedings of the Royal Society B, 283, 20152870 https://doi.org/10.1098/rspb.2015.2870 26842574 10.1098/rspb.2015.2870 PMC4760176

[ece34151-bib-0018] Halmetoja, A. , Valtonen, E. T. , & Taskinen, J. (1992). Trichodinids (Protozoa) on fish from four Central Finnish lakes of differing water quality. Aqua Fennica, 22, 59–70.

[ece34151-bib-0019] Hamilton, W. D. , & Zuk, M. (1982). Heritable true fitness and bright birds: A role for parasites? Science, 218, 384–387. https://doi.org/10.1126/science.7123238 7123238 10.1126/science.7123238

[ece34151-bib-0020] Hesthagen, I. H. (1977). Migrations, breeding and growth in *Pomatoschistus minutus* (Pallas) (Pisces, Gobiidae) in Oslofjorden, Norway. Sarsia, 63, 17–26. https://doi.org/10.1080/00364827.1977.10411316

[ece34151-bib-0021] Hõrak, P. , Ots, I. , Vellau, H. , Spottiswoode, C. , & Pape Møller, A. (2001). Carotenoid‐based plumage coloration reflects hemoparasite infection and local survival in breeding great tits. Oecologia, 126, 166–173. https://doi.org/10.1007/s004420000513 28547614 10.1007/s004420000513

[ece34151-bib-0022] Houde, A. E. , & Torio, A. J. (1992). Effect of parasitic infection on male color pattern and female choice in guppies. Behavioral Ecology, 3, 346–351. https://doi.org/10.1093/beheco/3.4.346

[ece34151-bib-0023] Järvenpää, M. , & Lindström, K. (2004). Water turbidity by algal blooms causes mating system breakdown in a shallow‐water fish, the sand goby *Pomatoschistus minutus* . Proceedings of the Royal Society B: Biological Sciences, 271, 2361–2365. https://doi.org/10.1098/rspb.2004.2870 15556888 10.1098/rspb.2004.2870 PMC1691863

[ece34151-bib-0024] Jokela, J. , Dybdahl, M. F. , & Lively, C. M. (2009). The maintenance of sex, clonal dynamics, and host‐parasite coevolution in a mixed population of sexual and asexual snails. American Naturalist, 174, S43–S53. https://doi.org/10.1086/599080 10.1086/599080 19441961

[ece34151-bib-0025] Jokela, J. , & Lively, C. M. (1995). Spatial variation in infection by digenetic trematodes in a population of fresh‐water snails (*Potamopyrgus antipodarum*). Oecologia, 103, 509–517. https://doi.org/10.1007/BF00328690 28307000 10.1007/BF00328690

[ece34151-bib-0026] Jokela, J. , Taskinen, J. , Mutikainen, P. , & Kopp, K. (2005). Virulence of parasites in hosts under environmental stress: Experiments with anoxia and starvation. Oikos, 108, 156–164. https://doi.org/10.1111/j.0030-1299.2005.13185.x

[ece34151-bib-0027] Karvonen, A. , Cheng, G. H. , & Valtonen, E. T. (2005). Within‐lake dynamics in the similarity of parasite assemblages of perch (*Perca fluviatilis*). Parasitology, 131, 817–823. https://doi.org/10.1017/S0031182005008425 16336735 10.1017/S0031182005008425

[ece34151-bib-0028] Karvonen, A. , Hudson, P. J. , Seppälä, O. , & Valtonen, E. T. (2004). Transmission dynamics of a trematode parasite: Exposure, acquired resistance and parasite aggregation. Parasitology Research, 92, 183–188. https://doi.org/10.1007/s00436-003-1035-y 14652746 10.1007/s00436-003-1035-y

[ece34151-bib-0029] Karvonen, A. , & Seehausen, O. (2012). The role of parasitism in adaptive radiations—When might parasites promote and when might they constrain ecological speciation? International Journal of Ecology, 2012, 1–20. https://doi.org/10.1155/2012/280169

[ece34151-bib-0030] Karvonen, A. , & Seppälä, O. (2008). Effect of eye fluke infection on the growth of whitefish (*Coregonus lavaretus*) – An experimental approach. Aquaculture, 279, 6–10. https://doi.org/10.1016/j.aquaculture.2008.04.013

[ece34151-bib-0031] Karvonen, A. , Seppälä, O. , & Valtonen, E. T. (2004a). Eye fluke‐induced cataract formation in fish: Quantitative analysis using an ophthalmological microscope. Parasitology, 129, 473–478. https://doi.org/10.1017/S0031182004006006 15521636 10.1017/s0031182004006006

[ece34151-bib-0032] Karvonen, A. , Seppälä, O. , & Valtonen, E. T. (2004b). Parasite resistance and avoidance behaviour in preventing eye fluke infections in fish. Parasitology, 129, 159–164. https://doi.org/10.1017/S0031182004005505 15376775 10.1017/s0031182004005505

[ece34151-bib-0033] King, K. C. , Delph, L. F. , Jokela, J. , & Lively, C. M. (2009). The geographic mosaic of sex and the red queen. Current Biology, 19, 1438–1441. https://doi.org/10.1016/j.cub.2009.06.062 19631541 10.1016/j.cub.2009.06.062

[ece34151-bib-0034] Klein, S. L. (2004). Hormonal and immunological mechanisms mediating sex differences in parasite infection. Parasite Immunology, 26, 247–264. https://doi.org/10.1111/j.0141-9838.2004.00710.x 15541029 10.1111/j.0141-9838.2004.00710.x

[ece34151-bib-0035] Koskivaara, M. , Valtonen, E. T. , & Prost, M. (1991). Dactylogyrids on the gills of roach in Central Finland – Features of infection and species composition. International Journal for Parasitology, 21, 565–572. https://doi.org/10.1016/0020-7519(91)90061-B 1743852 10.1016/0020-7519(91)90061-b

[ece34151-bib-0036] Lefevre, T. , Lebarbenchon, C. , Gauthier‐Clerc, M. , Misse, D. , Poulin, R. , & Thomas, F. (2009). The ecological significance of manipulative parasites. Trends in Ecology & Evolution, 24, 41–48. https://doi.org/10.1016/j.tree.2008.08.007 19026461 10.1016/j.tree.2008.08.007

[ece34151-bib-0037] Lindström, K. (1992). Female spawning patterns and male mating success in the sand goby *Pomatoschistus minutus* . Marine Biology, 113, 475–480. https://doi.org/10.1007/BF00349174

[ece34151-bib-0038] Lindström, K. (1998). Hietatokko In Raitaniemi J. (Ed.), Suomen luonto: Kalat, sammakkoeläimet ja matelijat (pp. 214). Helsinki, Finland: Weilin+Göös Oy.

[ece34151-bib-0039] Lindström, K. , & Lehtonen, T. K. (2013). Mate sampling and choosiness in the sand goby. Proceedings of the Royal Society B: Biological Sciences, 280, 1471–1476. 10.1098/rspb.2013.0983 PMC3712446 23804620

[ece34151-bib-0040] Lindström, K. , & Ranta, E. (1992). Predation by birds affects structure of breeding population in male sand gobies, *Pomatoschistus minutus* . Oikos, 64, 527–532. https://doi.org/10.2307/3545171

[ece34151-bib-0041] Lindström, K. , & Seppä, T. (1996). The environmental potential for polygyny and sexual selection in the sand goby, *Pomatoschistus minutus* . Proceedings of the Royal Society B: Biological Sciences, 263, 1319–1323. https://doi.org/10.1098/rspb.1996.0193

[ece34151-bib-0042] Lindström, K. , St. Mary, C. M. , & Pampoulie, C. (2006). Sexual selection for male parental care in the sand goby, *Pomatoschistus minutus* . Behavioral Ecology and Sociobiology, 60, 46–51. https://doi.org/10.1007/s00265-005-0138-0

[ece34151-bib-0043] Lopez, S. (1999). Parasitized female guppies do not prefer showy males. Animal Behaviour, 57, 1129–1134. https://doi.org/10.1006/anbe.1998.1064 10328800 10.1006/anbe.1998.1064

[ece34151-bib-0044] Louhi, K.‐R. , Karvonen, A. , Rellstab, C. , & Jokela, J. (2010). Is the population genetic structure of complex life cycle parasites determined by the geographic range of the most motile host? Infection, Genetics and Evolution, 10, 1271–1277. https://doi.org/10.1016/j.meegid.2010.08.013 10.1016/j.meegid.2010.08.013 20804859

[ece34151-bib-0045] Louhi, K.‐R. , Sundberg, L.‐R. , Jokela, J. , & Karvonen, A. (2015). Interactions among bacterial strains and fluke genotypes shape virulence of co‐infection. Proceedings of the Royal Society B: Biological Sciences, 282, 20152097 https://doi.org/10.1098/rspb.2015.2097 26674949 10.1098/rspb.2015.2097 PMC4707758

[ece34151-bib-0046] Maan, M. E. , van der Spoel, M. , Jimenez, P. Q. , van Alphen, J. J. M. , & Seehausen, O. (2006). Fitness correlates of male coloration in a Lake Victoria cichlid fish. Behavioral Ecology, 17, 691–699. https://doi.org/10.1093/beheco/ark020

[ece34151-bib-0047] Marcogliese, D. J. , Compagna, S. , Bergeron, E. , & McLaughlin, J. D. (2001). Population biology of eyeflukes in fish from a large fluvial ecosystem: The importance of gulls and habitat characteristics. Canadian Journal of Zoology, 79, 1102–1113. https://doi.org/10.1139/z01-077

[ece34151-bib-0048] Marcogliese, D. J. , Gendron, A. D. , Plante, C. , Fournier, M. , & Cyr, D. (2006). Parasites of spottail shiners (*Notropis hudsonius*) in the St. Lawrence River: Effects of municipal effluents and habitat. Canadian Journal of Zoology, 84, 1461–1481. https://doi.org/10.1139/z06-088

[ece34151-bib-0049] Mazzi, D. (2004). Parasites make male pipefish careless. Journal of Evolutionary Biology, 17, 519–527. https://doi.org/10.1111/j.1420-9101.2004.00704.x 15149395 10.1111/j.1420-9101.2004.00704.x

[ece34151-bib-0050] McGraw, K. J. , & Hill, G. E. (2000). Differential effects of endoparasitism on the expression of carotenoid‐ and melanin‐based ornamental coloration. Proceedings of the Royal Society B: Biological Sciences, 267, 1525–1531. https://doi.org/10.1098/rspb.2000.1174 11007328 10.1098/rspb.2000.1174 PMC1690705

[ece34151-bib-0051] Moore, J. (2002). Parasites and the behavior of animals. New York, NY: Oxford University Press.

[ece34151-bib-0052] Nyman, K.‐J. (1953). Observations on the behaviour of *Gobius microps* . Acta Societatis pro Fauna Flora Fennica, 69, 1–11.

[ece34151-bib-0053] Owen, S. F. , Barber, I. , & Hart, P. J. B. (1993). Low‐level infection by eye fluke, *Diplostomum* spp, affects the vision of 3‐spined sticklebacks, *Gasterosteus aculeatus* . Journal of Fish Biology, 42, 803–806. https://doi.org/10.1111/j.1095-8649.1993.tb00387.x

[ece34151-bib-0054] Pampoulie, C. , Lindström, K. , & St. Mary, C. M. (2004). Have your cake and eat it too: Male sand gobies show more parental care in the presence of female partners. Behavioral Ecology, 15, 199–204. https://doi.org/10.1093/beheco/arg107

[ece34151-bib-0055] Pfennig, K. S. , & Tinsley, R. C. (2002). Different mate preferences by parasitized and unparasitized females potentially reduces sexual selection. Journal of Evolutionary Biology, 15, 399–406. https://doi.org/10.1046/j.1420-9101.2002.00406.x

[ece34151-bib-0056] Poulin, R. (1996). Helminth growth in vertebrate hosts: Does host sex matter? International Journal for Parasitology, 26, 1311–1315. https://doi.org/10.1016/S0020-7519(96)00108-7 9024877 10.1016/s0020-7519(96)00108-7

[ece34151-bib-0057] Poulin, R. , & Vickery, W. L. (1996). Parasite mediated sexual selection: Just how choosy are parasitized females? Behavioral Ecology and Sociobiology, 38, 43–49. https://doi.org/10.1007/s002650050215

[ece34151-bib-0058] Richards, E. L. , van Oosterhout, C. , & Cable, J. (2010). Sex‐specific differences in shoaling affect parasite transmission in guppies. PLoS ONE, 5, e13285 https://doi.org/10.1371/journal.pone.0013285 20949014 10.1371/journal.pone.0013285 PMC2952601

[ece34151-bib-0059] Rintamäki‐Kinnunen, P. , & Valtonen, E. T. (1997). Epizootiology of protozoans in farmed salmonids at northern latitudes. International Journal for Parasitology, 27, 89–99. https://doi.org/10.1016/S0020-7519(96)00162-2 9076534 10.1016/s0020-7519(96)00162-2

[ece34151-bib-0060] de Roij, J. , & MacColl, A. D. C. (2012). Consistent differences in macroparasite community composition among populations of three‐spined sticklebacks, *Gasterosteus aculeatus* L. Parasitology, 139, 1478–1491. https://doi.org/10.1017/S0031182012000789 23025902 10.1017/S0031182012000789

[ece34151-bib-0061] Seehausen, O. , van Alphen, J. J. M. , & Witte, F. (1997). Cichlid fish diversity threatened by eutrophication that curbs sexual selection. Science, 277, 1808–1811. https://doi.org/10.1126/science.277.5333.1808

[ece34151-bib-0062] Seppälä, O. , Karvonen, A. , & Valtonen, E. T. (2005). Manipulation of fish host by eye flukes in relation to cataract formation and parasite infectivity. Animal Behaviour, 70, 889–894. https://doi.org/10.1016/j.anbehav.2005.01.020

[ece34151-bib-0063] Seppälä, O. , Liljeroos, K. , Karvonen, A. , & Jokela, J. (2008). Host condition as a constraint for parasite reproduction. Oikos, 117, 749–753. https://doi.org/10.1111/j.0030-1299.2008.16396.x

[ece34151-bib-0064] Sikkel, P. C. , Fuller, C. A. , & Hunte, W. (2000). Habitat/sex differences in time at cleaning stations and ectoparasite loads in a Caribbean reef fish. Marine Ecology Progress Series, 193, 191–199. https://doi.org/10.3354/meps193191

[ece34151-bib-0065] Sikkel, P. C. , Nemeth, D. , McCammon, A. , & Williams, E. H. (2009). Habitat and species differences in prevalence and intensity of *Neobenedenia melleni* (Monogenea: Capsalidae) on sympatric Caribbean surgeonfishes (Acanthuridae). Journal of Parasitology, 95, 63–68. https://doi.org/10.1645/GE-1645.1 18684015 10.1645/GE-1645.1

[ece34151-bib-0066] Stephenson, J. F. , van Oosterhout, C. , Mohammed, R. S. , & Cable, J. (2015). Parasites of Trinidadian guppies: Evidence for sex‐ and age‐specific trait‐mediated indirect effects of predators. Ecology, 96, 489–498. https://doi.org/10.1890/14-0495.1 26240870 10.1890/14-0495.1

[ece34151-bib-0067] Tadiri, C. P. , Scott, M. E. , & Fussmann, G. F. (2016). Impact of host sex and group composition on parasite dynamics in experimental populations. Parasitology, 143, 523–531. https://doi.org/10.1017/S0031182016000172 26888157 10.1017/S0031182016000172

[ece34151-bib-0068] Taskinen, J. , Valtonen, E. T. , & Mäkelä, T. (1994). Quantity of sporocysts and seasonality of two *Rhipidocotyle* species (Digenea: Bucephalidae) in *Anodonta piscinalis* (Mollusca: Bivalvia). International Journal for Parasitology, 24, 877–886. https://doi.org/10.1016/0020-7519(94)90014-0 7982750 10.1016/0020-7519(94)90014-0

[ece34151-bib-0069] Thompson, C. W. , Hillgarth, N. , Leu, M. , & McClure, H. E. (1997). High parasite load in house finches (*Carpodacus mexicanus*) is correlated with reduced expression of a sexually selected trait. American Naturalist, 149, 270–294. https://doi.org/10.1086/285990

[ece34151-bib-0070] Tompkins, D. M. , Dunn, A. M. , Smith, M. J. , & Telfer, S. (2011). Wildlife diseases: From individuals to ecosystems. Journal of Animal Ecology, 80, 19–38. https://doi.org/10.1111/j.1365-2656.2010.01742.x 20735792 10.1111/j.1365-2656.2010.01742.x

[ece34151-bib-0071] Van Damme, P. A. , & Ollevier, F. (1994). Infection dynamics of *Lernaeocera lusci* on sand goby *Pomatoschistus minutus* in the Oosterschelde (The Netherlands). Diseases of Aquatic Organisms, 19, 83–87. https://doi.org/10.3354/dao019083

[ece34151-bib-0072] Wood, C. L. , Byers, J. E. , Cottingham, K. L. , Altman, I. , Donahue, M. J. , & Blakeslee, A. M. H. (2007). Parasites alter community structure. Proceedings of the National Academy of Sciences of the United States of America, 104, 9335–9339. https://doi.org/10.1073/pnas.0700062104 17517667 10.1073/pnas.0700062104 PMC1890495

[ece34151-bib-0073] Zander, C. D. (2003). Four‐year monitoring of parasite communities in gobiid fishes of the south‐western Baltic – I. Guild and component community. Parasitology Research, 90, 502–511. https://doi.org/10.1007/s00436-003-0887-5 12827504 10.1007/s00436-003-0887-5

[ece34151-bib-0074] Zander, C. D. (2005). Four‐year monitoring of parasite communities in gobiid fishes of the southwest Baltic. Parasitology Research, 95, 136–144. https://doi.org/10.1007/s00436-004-1252-z 15609062 10.1007/s00436-004-1252-z

[ece34151-bib-0075] Zander, C. D. , & Kesting, V. (1998). Colonization and seasonality of goby (Gobiidae, Teleostei) parasites from the southwestern Baltic Sea. Parasitology Research, 84, 459–466. https://doi.org/10.1007/s004360050430 9660135 10.1007/s004360050430

[ece34151-bib-0076] Zander, C. D. , Strohbach, U. , & Groenewold, S. (1993). The importance of gobies (Gobiidae, Teleostei) as hosts and transmitters of parasites in the SW Baltic. Helgolander Meeresuntersuchungen, 47, 81–111. https://doi.org/10.1007/BF02366186

